# Editorial: Signaling pathways behind immune evasion and therapy resistance

**DOI:** 10.3389/fimmu.2022.1104167

**Published:** 2022-12-09

**Authors:** Elena Martin-Orozco, Lisheng Wang, Shilpak Chatterjee, Brent A. Hanks

**Affiliations:** ^1^ Murcia BioHealth Research Institute, University of Murcia, Murcia, Spain; ^2^ Department of Biochemistry, Microbiology, and Immunology, University of Ottawa, Ottawa, ON, Canada; ^3^ Indian Institute of Chemical Biology (CSIR), Kolkata, India; ^4^ Department of Medicine, Duke University, Durham, NC, United States

**Keywords:** tumor immunity, PD-L1, cAMP, UPR, PDE4B, N6-methyladenosine, UQCRC1, PDIA5

Immune evasion strategies and therapy resistance are two decisive mechanisms that correlate with patient prognosis and response to treatment in many cancer types. These are processes controlled by a net of interrelated and complex signaling pathways involved in cell survival, tumor metastases. as well as inflammatory responses. New strategies in tumor therapy have been developed, such as the use of immune check point inhibitors (ICI), especially those targeting PD-1/PD-L1 and others; nevertheless, they have been shown to be effective only in select subsets of patients while others remain unresponsive. This highlights the need to discover new therapeutic targets and prognostic biomarkers for cancer treatment.

In this issue, the authors propose new biomarkers and/or therapeutic targets based on signaling pathways with a role in cellular homeostasis, such as the cAMP pathway and the unfolded protein response (UPR) pathway. The cAMP molecule is a well-known regulator of innate and adaptive immunity. Molecules that elevate intracellular cAMP levels inhibit the synthesis of pro-inflammatory molecules and induce the production of anti-inflammatory factors in a broad range of immune cells. As part of the immunosuppressive actions mediated by cAMP, PD-L1 over-expression has been described in normal and malignant lymphocytes (1). Additionally, the Unfolded Protein Response (UPR) is a complex network of molecules that under physiological conditions controls protein folding fidelity in the endoplasmic reticulum to maintain protein homeostasis (proteostasis). Disrupted ER proteostasis could lead to tumorigenesis and altered anti-tumoral immunity since there is a connection at many levels between this pathway, tumor survival, and immune cell differentiation and function (2).

New insights into the role of the molecules involved in these pathways underscore their importance in cancer and anti-tumoral immunity by controlling the expression of checkpoint inhibitors such as PD-L1, immune infiltration, effector functions of NK cells, and others.

Tong et al., demonstrate that the protein cyclic adenosine monophosphate phosphodiesterase 4B (PDE4B), which degrades intracellular cyclic nucleotides, has a protective role by increasing anti-tumor immunity. Thus, these researchers identified a regulatory effect of the cAMP/PDE4B pathway on immune infiltration and tumor prognosis in Lung Adenocarcinoma (LUAD). In addition, they have demonstrated the existence of a regulatory network between the cAMP/PDE4 axis and the expression of PD-L1, which has important implications regarding the clinical management of ICI-based immunotherapy in this tumor type (1, 3).

Another potential cancer biomarker described by Chen et al. is N6-methyladenosine, a modified nucleoside that originates from RNA degradation and after being released into the extracellular space, can act as a signaling molecule. Specifically, this molecule regulates physiological functions *via* their binding to certain receptors on the cell membrane, such as the Adenosine A3 receptor, inducing cAMP downregulation (4). Tracking this molecule, Chen et al. have identified up to 18 N6-methyladenosine regulated genes that are abnormally expressed in Head and Neck Squamous Cell Carcinoma compared with normal tissues. These researchers classified patients as high or low risk, depending on the abnormal expression of 12 of these genes and further correlate low risk ICI-treated patients with an increase in objective response rate as well as overall survival. Finally, they found low risk patients to exhibit an increase in CD8 T cell, Treg, and T_FH_ infiltration within the tumor microenvironment (TME).

A third molecule with cAMP regulatory activity is the ubiquinol-cytochrome c reductase core protein I (UQCRC1). This protein is a key component of the mitochondrial complex III and participates in oxidative phosphorylation, increasing ATP and cAMP levels and inducing adenosine generation (5). Accordingly, Cong et al. demonstrate that increased expression of UQCRC1 in pancreatic cancer contributes to immune evasion by inhibiting the recruitment and cytotoxic activity of NK cells within the TME. Additionally, these authors found diminished levels of CCL5 expression by cancer cells as well as a shift towards inhibitory receptors, such as CD96, in NK cells.

Finally, Chen et al. reports disulfide isomerase A5 (PDIA5), an endoplasmic reticulum protein involved in the UPR pathway to be aberrantly expressed in several human cancers. Importantly, these authors demonstrated PDIA5 expression to correlate with decreased PD-L1 expression and immune cell infiltration, specifically M2 macrophages within the TME. Hence, this protein fulfills the dual function of being a modulator of proteostasis, but also a potential biomarker of cancer.

In summary, the articles presented in this issue describe molecules involved in biological processes that support cellular homeostasis and that, in the context of altered expression in a tumor cell contribute to disease progression by impairing anti-tumoral immunity and driving resistance to immunotherapy. Further investigation of these proteins promises to reveal new insight into these immune evasion mechanisms and the discovery of novel immunotherapeutic targets and biomarkers capable of improving the management of cancer patients.

## Author contributions

Drafting of manuscript: EM-O. Critical revision and/or final approval of the version to be published: BH, LW, SC and EM-O.
